# Associations of Ultra‐Processed Food Consumption and Night Eating With GI Symptom‐Specific Anxiety in Reproductive‐Age Women With Polycystic Ovary Syndrome

**DOI:** 10.1002/fsn3.72007

**Published:** 2026-06-10

**Authors:** Emine Merve Ekici, Sena Dilşad Akçakaya, Vefa Şakar, Özge Mengi Çelik

**Affiliations:** ^1^ Department of Nutrition and Dietetics, Gülhane Health Sciences Faculty University of Health Sciences Ankara Turkey; ^2^ Department of Nutrition and Dietetics, Gülhane Health Sciences Institute University of Health Sciences Ankara Turkey; ^3^ Department of Obstetrics and Gynecology Gülhane Training and Research Hospital Ankara Turkey

**Keywords:** behavioral nutrition, gastrointestinal‐specific anxiety, night eating symptoms, polycystic ovary syndrome, ultra processed food

## Abstract

Polycystic ovary syndrome (PCOS) is a complex endocrine disorder often accompanied by metabolic disturbances, night eating patterns, and gastrointestinal‐related distress. This cross‐sectional study examined the associations between ultra‐processed food (UPF) consumption, night eating symptoms, and gastrointestinal (GI) symptom‐specific anxiety in 205 women (aged 18–50 years) with a physician‐confirmed PCOS diagnosis. Participants completed validated questionnaires assessing UPF consumption (sQ‐HPF), the severity of night eating symptoms (NEQ), and GI‐specific anxiety (VSI), alongside self‐reported anthropometric measurements. Multiple linear regression models were used to identify independent associations, adjusting for body mass index (BMI), smoking status, and socioeconomic status. While higher UPF consumption was significantly associated with increased NEQ scores and higher GI‐specific anxiety in bivariate analyses, its independent influence was attenuated in final adjusted regression models. In these adjusted models, GI‐specific anxiety and BMI were independently associated with NEQ scores (*p* < 0.05), while night eating symptoms emerged as a primary factor associated with GI‐specific anxiety (*p* < 0.001). Sensitivity analyses, including subgroup analysis for BMI < 35 kg/m^2^ and multivariate models excluding BMI as a covariate, confirmed that the observed associations remained robust. These findings suggest a behavioral and dietary interplay where UPF consumption and night eating symptoms are linked to GI‐specific anxiety in women with PCOS. Considering that lifestyle modification is a cornerstone of PCOS management, integrating the assessment of dietary patterns and night eating into routine care may support a more comprehensive approach. Future longitudinal studies are warranted to clarify the directional nature of these associations linking UPF exposure, night eating symptoms, and psychological GI distress in this population.

## Graphical abstract

This cross‐sectional study investigated the relationships between ultra‐processed food (UPF) consumption, night eating symptoms, and gastrointestinal (GI)‐specific anxiety in 205 women with polycystic ovary syndrome (PCOS). UPF consumption was associated with GI‐specific anxiety in unadjusted analyses but was not an independent predictor after adjustment for BMI, smoking status, and socioeconomic status. Night eating symptoms were independently associated with GI‐specific anxiety, while both GI‐specific anxiety and BMI independently predicted night eating symptoms. These findings highlight the importance of eating behaviors and psychological GI distress in women with PCOS and suggest that comprehensive assessment of dietary patterns and night eating symptoms may support improved clinical management.

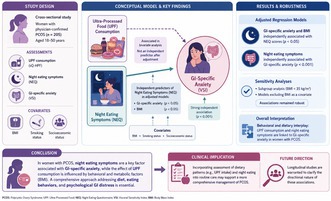

**Abbreviations**
BMI: Body Mass Index
GI: gastrointestinal
IBS: irritable bowel syndrome
NEQ: Night Eating Questionnaire
NES: Night Eating Syndrome
PCOS: Polycystic Ovary Syndrome
sQ‐HPF: Screening Questionnaire of Highly Processed Food Consumption
UPF: ultra‐processed food
VSI: Visceral Sensitivity Index

## 1 Introduction

Polycystic ovary syndrome (PCOS) is a complex endocrine and metabolic disorder characterized by hyperandrogenism, ovulatory dysfunction, and chronic low‐grade inflammation (Rodriguez Paris and Bertoldo 2019). Beyond reproductive impairments, PCOS presents a broad metabolic phenotype where obesity and insulin resistance are frequently implicated, often exacerbated by disordered eating behaviors (Gambineri et al. 2002; The Thessaloniki ESHRE/ASRM‐Sponsored PCOS Consensus Workshop Group 2008). Consequently, although lifestyle modification remains the cornerstone of management, there is a growing need to shift the focus from traditional caloric restriction to the qualitative and behavioral aspects of nutrition to mitigate chronic complications effectively (Garvey et al. 2016).

In recent years, dietary patterns have shifted toward the frequent consumption of highly processed foods (HPF), often referred to in broader contexts as ultra‐processed foods (UPF), which are characterized by high energy density, refined carbohydrates, and low nutritional value (Monteiro et al. 2018). High UPF intake is associated with obesity, insulin resistance, and systemic inflammation, all of which are central to the PCOS phenotype (Askari et al. 2020). Previous evidence suggests that women with PCOS may consume higher amounts of these products, potentially influencing symptom severity through mechanisms such as the accumulation of advanced glycation end products (Barrea et al. 2019; Merhi 2019; Szczuko et al. 2016). Despite these potential risks, the nutritional implications of UPF exposure in this population remain underexplored, with only a few studies specifically examining these consumption patterns (Ayton et al. 2021; Derrigo and LaFata 2023; Hajivandi et al. 2020).

Beyond metabolic and reproductive concerns, PCOS significantly impacts gastrointestinal (GI) function. Women with PCOS report a high prevalence of GI complaints and functional disorders such as irritable bowel syndrome (IBS), often linked to hormonal imbalances and elevated stress levels (Kałużna et al. 2022; Martin et al. 2017; Mulak et al. 2014). More recently, these symptoms have been conceptualized within the framework of GI symptom‐specific anxiety, which reflects a cognitive‐affective hypersensitivity and fear of GI sensations rather than purely physiological nociception (Peters et al. 2022). Given the activation of the hypothalamic–pituitary–adrenal axis and increased perceived stress in women with PCOS, this psychological burden of GI symptoms may play a critical role in the clinical presentation of the syndrome (Martin et al. 2017). However, its potential link to specific dietary patterns remains largely unexamined.

The metabolic and psychological burden of PCOS often manifests as disruptions in eating patterns. Night eating symptoms, characterized by nocturnal hyperphagia or morning anorexia, have been linked to circadian disruptions and stress‐induced eating in this population (Davis et al. 2011; Olszanecka‐Glinianowicz et al. 2020; Ozgen Saydam and Yildiz 2021). While the Night Eating Questionnaire (NEQ) is widely used to assess the severity of these symptoms and the likelihood of Night Eating Syndrome (NES) (Allison et al. 2008), its relationship with specific dietary qualities remains unclear. International guidelines emphasize meal timing and nutritional quality as cornerstones of PCOS care (Teede et al. 2018, 2023); however, the potential interplay between UPF consumption, night eating symptoms, and GI‐specific anxiety has not been investigated concurrently. Understanding these clinical associations is essential for developing integrated, symptom‐focused nutritional interventions. Therefore, examining the relationships between UPF consumption, GI‐specific anxiety, and the severity of night eating may provide insight into the clinical and dietary links relevant to PCOS management.

The conceptual framework of this study is anchored in a dietary‐behavioral‐psychological triad. In this model, we propose that UPF consumption is associated with metabolic and reward‐system dysregulation, which may relate to the development of night eating symptoms. Subsequently, the psychological distress of maladaptive eating patterns is linked to the cognitive‐affective response of GI‐specific anxiety. Therefore, we hypothesized that higher UPF consumption and increased night eating symptoms would be positively associated with higher levels of GI‐specific anxiety in women with PCOS.

This study aims to evaluate the relationships between UPF consumption, GI‐specific anxiety, and night eating in women with PCOS. Understanding these associations may support the development of integrated dietary recommendations and behavioral interventions for PCOS, while contributing to filling the literature gap regarding the impact of UPF exposure in this clinical population.

## 2 Materials and Methods

### 2.1 Study Design and Ethics

This cross‐sectional study was conducted between September 2024 and January 2025 on 205 women (aged 18–50 years) diagnosed with PCOS. Ethical approval was obtained from the University of Health Sciences Gülhane Scientific Research Ethics Committee (Decision No: 2024/547). All procedures were performed in accordance with the Declaration of Helsinki. Prior to participation, written informed consent was obtained from all participants through a physical signature on a formal consent form during their clinical visit.

### 2.2 Participants and Recruitment

Inclusion criteria were voluntary participation, age between 18 and 50 years, physician‐confirmed diagnosis of PCOS by an obstetrician and gynecologist according to the Rotterdam criteria, no oral contraceptive use in the last 3 months, and not being pregnant or breastfeeding. Exclusion criteria included: diagnosis of type 2 diabetes mellitus, Cushing's syndrome, thyroid disorders, or other endocrine abnormalities; history of bariatric or major GI surgery; active psychiatric disorders; and regular use of medications affecting appetite, GI motility, or reproductive hormones (e.g., metformin, SSRIs, SNRIs). Participants with chronic GI diseases such as IBD or celiac disease were also excluded. PCOS diagnosis required at least two of the following: oligo‐anovulation (menstrual cycles > 35 days or < 9 periods/year), clinical and/or biochemical hyperandrogenism (Ferriman‐Gallwey score ≥ 8 or persistent acne), and polycystic ovaries on ultrasound. All participants were recruited from the Department of Obstetrics and Gynecology at Gülhane Training and Research Hospital and referred through a structured appointment system to ensure consistency.

### 2.3 Procedure and Consent Sequence

Data collection was conducted through face‐to‐face, supervised interviews in a controlled clinical setting. The process followed a standardized protocol: initially, participants reviewed and signed a physical, written informed consent form. Following the acquisition of the handwritten signature, a tablet was provided for survey completion. At the commencement of the digital interface, participants also marked a digital checkbox as a supervised confirmation. This dual‐consent approach verified that the module functioned as a supervised session rather than an open‐access online survey, thereby minimizing selection and coverage biases. A copy of the Informed Consent Form used in this study is provided in Appendix S1.

### 2.4 Data Collection

The structured questionnaire covered demographic characteristics (age, education, income), lifestyle factors (smoking, alcohol), and PCOS‐related health information (time since diagnosis). Validated psychometric scales were used to assess the consumption of UPF (sQ‐HPF), the severity of night eating symptoms (NEQ), and GI symptom‐specific anxiety (VSI). Despite the face‐to‐face format, anthropometric measurements (weight and height) were obtained via self‐report. This approach was preferred to maintain a standardized protocol and to minimize potential participant discomfort or psychological distress/weight stigma associated with on‐site weighing in a clinical environment. Participants were provided with detailed written instructions to ensure accurate self‐reporting.

### 2.5 Anthropometric Measurements

Anthropometric measurements, including body weight and height, were obtained via standardized self‐report. To ensure data reliability and minimize reporting bias, participants were provided with detailed, standardized written instructions for accurate measurement techniques. BMI was calculated using the standard formula: weight (kg)/height^2^ (m^2^). Weight status was categorized according to the World Health Organization (WHO) criteria: BMI < 18.50 kg/m^2^ was classified as underweight, 18.50–24.99 kg/m^2^ as normal weight, 25.00–29.99 kg/m^2^ as overweight, and ≥ 30.00 kg/m^2^ as obese (WHO 2025).

### 2.6 Screening Questionnaire of Highly Processed Food Consumption (sQ‐HPF)

The sQ‐HPF was developed by Martinez‐Perez et al. (2022) and validated for the Turkish population by Erdoğan Gövez et al. (2024), was used to provide a screening score for UPF consumption. The sQ‐HPF was used specifically to provide a screening score of UPF consumption, rather than a precise quantification of ultra‐processed food intake as a percentage of total energy. The scale consists of 11 food items scored dichotomously (1 = yes; 0 = no), with total scores ranging from 0 to 11. A score ≥ 6 was used as the cutoff to indicate high UPF consumption, while scores < 6 reflected low UPF consumption. The Cronbach's alpha coefficient for the scale was reported as 0.65. The sQ‐HPF showed a Cronbach's alpha of 0.61, which is considered satisfactory for this population.

### 2.7 Night Eating Questionnaire (NEQ)

The severity of NES symptoms was assessed using the NEQ, a 14‐item screening instrument developed by (Allison et al. 2008). The NEQ evaluates the core clinical features of NES, including evening hyperphagia, morning anorexia, and nocturnal ingestions. Each item is scored on a Likert‐type scale from 0 to 4, with total scores ranging from 0 to 52. Higher scores indicate greater symptom severity, and a total score of ≥ 25 is considered the threshold for identifying individuals likely to meet the diagnostic criteria for NES. The Turkish validity and reliability study was conducted by Atasoy et al. (2014), who reported a Cronbach's alpha coefficient of 0.69. In the current sample, the NEQ showed strong internal consistency with a Cronbach's alpha of 0.80.

### 2.8 Visceral Sensitivity Index (VSI)

GI symptom‐specific anxiety was evaluated using the VSI, a 15‐item unidimensional scale developed by Labus et al. (2004) to measure GI symptom‐specific anxiety. This instrument captures the cognitive, affective, and behavioral dimensions of fear related to GI symptoms. Items are rated on a six‐point Likert scale (0 = strongly disagree to 5 = strongly agree), with total scores ranging from 0 to 75. Higher scores indicate greater GI‐specific anxiety (frequently referred to as visceral sensitivity in clinical contexts). The original study reported a Cronbach's alpha of 0.93, and the Turkish validity and reliability study was performed by Abay (2024). The VSI demonstrated excellent internal consistency in this study with a Cronbach's alpha of 0.95.

### 2.9 Statistical Analysis

Statistical analyses were performed using IBM SPSS Statistics for Windows (version 27.0). AI‐based tools were utilized solely for linguistic refinement and graphical visualization; all scientific interpretations were conducted by the authors. Data distribution was evaluated through histograms, Q–Q plots, and the Shapiro–Wilk test, complemented by skewness and kurtosis coefficients. Although certain variables (specifically NEQ and BMI) showed significant deviations from normality (*p* < 0.05), parametric procedures (Independent *t*‐tests, Pearson's correlation, and linear regression) were prioritized due to the robustness provided by the sample size (*N* = 205) according to the Central Limit Theorem. To ensure the reliability of the findings, non‐parametric sensitivity analyses (Spearman's correlation and Mann–Whitney *U* tests) were also performed. Continuous variables were expressed as mean ± standard deviation (SD), and categorical variables as frequencies and percentages. Multiple linear regression models were constructed to identify independent associations with (i) the severity of night eating (NEQ total score) and (ii) GI symptom‐specific anxiety (VSI total score) as dependent variables. Models were adjusted for potential confounders, including BMI, smoking status, alcohol consumption, marital status, and income level. Categorical independent variables were included in the linear regression models as dummy‐coded variables. Specifically, marital status (reference: single), income status (reference: low income), and smoking status (reference: non‐smoker) were transformed into binary variables. Income status was treated as an ordinal variable in the models. This coding approach allowed for a clear interpretation of the beta coefficients relative to the specified reference groups. The selection of independent variables for the regression models was informed by a theoretical framework based on existing literature in the fields of PCOS and eating disorders. BMI, smoking status, and socioeconomic factors (income and marital status) were included as covariates because they are well‐established confounders associated with both metabolic health and maladaptive eating patterns (Greenwood et al. 2020; Sutcu et al. 2021). GI symptom‐specific anxiety (VSI scores) and UPF consumption were selected as primary predictors to test our core hypothesis regarding the psychological and dietary drivers of night eating symptoms in women with PCOS. Standardized beta coefficients (*beta*), *t*‐values, and *p*‐values were reported, with model fit assessed via *R* ^2^. Effect sizes were reported and interpreted using standardized beta coefficients and *R* ^2^ values for regression models, and Cohen's *d* for group comparisons, with magnitudes interpreted according to established benchmarks (small: 0.2, medium: 0.5, large: 0.8 for *d*; small: 0.02, medium: 0.13, large: 0.26 for *R* ^2^). To minimize Type I error risks associated with multiplicity, core associations were primarily interpreted at significance levels of *p* < 0.01 and *p* < 0.001. For all other analyses, a *p*‐value of < 0.05 was considered statistically significant. Finally, to account for potential systematic reporting bias in self‐reported anthropometrics, Pearson correlation analyses were repeated in a subgroup of participants with BMI < 35 kg/m^2^ (*n* = 182) as a robustness check (summary Table 1).

**TABLE 1:** General characteristics of individuals.

Variables	X¯ ± SD
Age (years)	25.89 ± 3.91
Duration since PCOS diagnosis (years)	6.08 ± 4.44
Weight (kg)	72.33 ± 19.44
BMI (kg/m^2^)	26.98 ± 6.51
Screening Questionnaire of Highly Processed Food Consumption total score	6.44 ± 2.82
Night Eating Questionnaire Total Score	19.24 ± 9.20
Visceral Sensitivity Index total score	41.12 ± 16.29
	**Number (%)**
Education level	
Middle school	1 (0.5)
High school	4 (2.0)
University	173 (84.4)
Master's degree/doctorate	27 (13.2)
Occupation	
Housewife	14 (6.8)
Officer	60 (29.3)
Student	60 (29.3)
Private sector employee	30 (14.6)
Worker	13 (6.3)
Unemployed	28 (13.7)
Marital status	
Single	118 (57.6)
Married	87 (42.4)
Having children	
Yes	20 (9.8)
No	185 (90.2)
Income status	
Income less than expenses	58 (28.3)
Income equal to expenses	38 (18.5)
Income more than expenses	109 (53.2)
Smoking	
Yes	50 (24.4)
No	155 (75.6)
Alcohol consumption	
Yes	52 (25.4)
No	153 (74.6)
Presence of disease	
Yes	77 (37.6)
No	128 (62.4)
Symptoms of PCOS^a^	
Menstrual irregularity	165 (80.5)
Hirsutism	165 (80.5)
Acne	138 (67.3)
Infertility	20 (9.8)
Obesity	77 (37.6)
Hair loss	133 (64.9)
Fatigue	189 (92.2)
BMI classification	
Underweight	7 (3.4)
Normal	87 (42.4)
Overweight	57 (27.8)
Obese	53 (25.9)

*Note:* Mean ± standard deviation (X¯ ± SD) for continuous variables and as number (percentage) [*n* (%)] for categorical variables.

Abbreviations: BMI, body mass index; NEQ, Night Eating Questionnaire; PCOS, Polycystic Ovary Syndrome; sQ‐HPF, Screening Questionnaire of Highly Processed Food Consumption; VSI, Visceral Sensitivity Index.

^a^ Participants were able to select more than one symptom; therefore, percentages may exceed 100% for this category.

Prior to conducting the linear regression analyses, the necessary statistical assumptions were verified. Multicollinearity was assessed using Variance Inflation Factors (VIF); all VIF values were found to be below 2.0 (well within the acceptable limit of < 10), indicating no significant multicollinearity issues. Normality of residuals was confirmed through visual inspection of P–P plots and histograms. Furthermore, homoscedasticity was verified by examining residual scatter plots, which demonstrated a random distribution of errors, and the independence of observations was confirmed via the Durbin–Watson test (values ranged between 1.5 and 2.5). To facilitate a clearer interpretation of the relative impact of predictors, the standardized beta coefficients from the multiple linear regression models were visualized using bar charts (Figure 2a,b).

To evaluate the robustness of our findings and address the potential for systematic bias in self‐reported anthropometric data, we performed sensitivity analyses. Specifically, we re‐ran the multivariate linear regression models excluding BMI as a covariate to ensure the stability of the observed associations. Additionally, the robustness of the primary correlations was examined within a subgroup of participants with BMI < 35 kg/m^2^ (*n* = 182).

## 3 Results

Table 1 presents the general characteristics of the women who participated in the study. The study included 205 women with a mean age of 25.89 ± 3.91 years and a mean PCOS diagnosis duration of approximately 6 years. The majority of the participants were highly educated (97.6% university degree or higher) and the sample was predominantly composed of non‐smokers and non‐drinkers. Regarding PCOS‐related clinical presentations, fatigue (92.2%), excessive hair growth (80.5%), and menstrual irregularities (80.5%) were the most prevalent symptoms. While 37.6% of participants reported a history of obesity since their diagnosis, the current obesity prevalence was 25.9%, suggesting weight fluctuations over time.

Table 2 presents the dietary habits of the participants. It was found that 69.8% of the women skipped main meals, while 54.1% consumed regular snacks. In terms of eating out, 2.9% ate outside every day, whereas 39.5% ate outside once or twice a week. Additionally, 34.6% of the participants were categorized as having a high level of UPF consumption based on the sQ‐HPF cutoff score. The prevalence of NES symptoms among the participants was 21%.

**TABLE 2:** Characteristics of individuals' eating habits.

Variables	Number (%)
Meal skipping	
Yes	143 (69.8)
No	62 (30.2)
Regular snack	
Yes	111 (54.1)
No	94 (45.9)
Frequency of eating out of the house	
None	5 (2.4)
Every day	6 (2.9)
Once or twice a week	81 (39.5)
Three or four times a week	30 (14.6)
Five or six times a week	10 (4.9)
Once or twice a month	73 (35.6)
Frequency of consumption of sugar‐containing foods	
None	2 (1)
Every day	97 (47.3)
Once or twice a week	39 (19.0)
Three or four times a week	42 (20.5)
Five or six times a week	19 (9.3)
Once or twice a month	6 (2.9)
Ultra processed food consumption^a^	
High Consumption	71 (34.6)
Low Consumption	134 (65.4)
Night eating syndrome^b^	
Yes	43 (21.0)
No	162 (79.0)

^a^ Ultra‐processed food (UPF) consumption categories were determined using the Screening Questionnaire of Highly Processed Food Consumption (sQ‐HPF), where a score of ≥ 6 indicates high consumption.

^b^ Night Eating Syndrome status represents the likelihood of meeting diagnostic criteria based on a Night Eating Questionnaire total score of ≥ 25.

**FIGURE 1:**
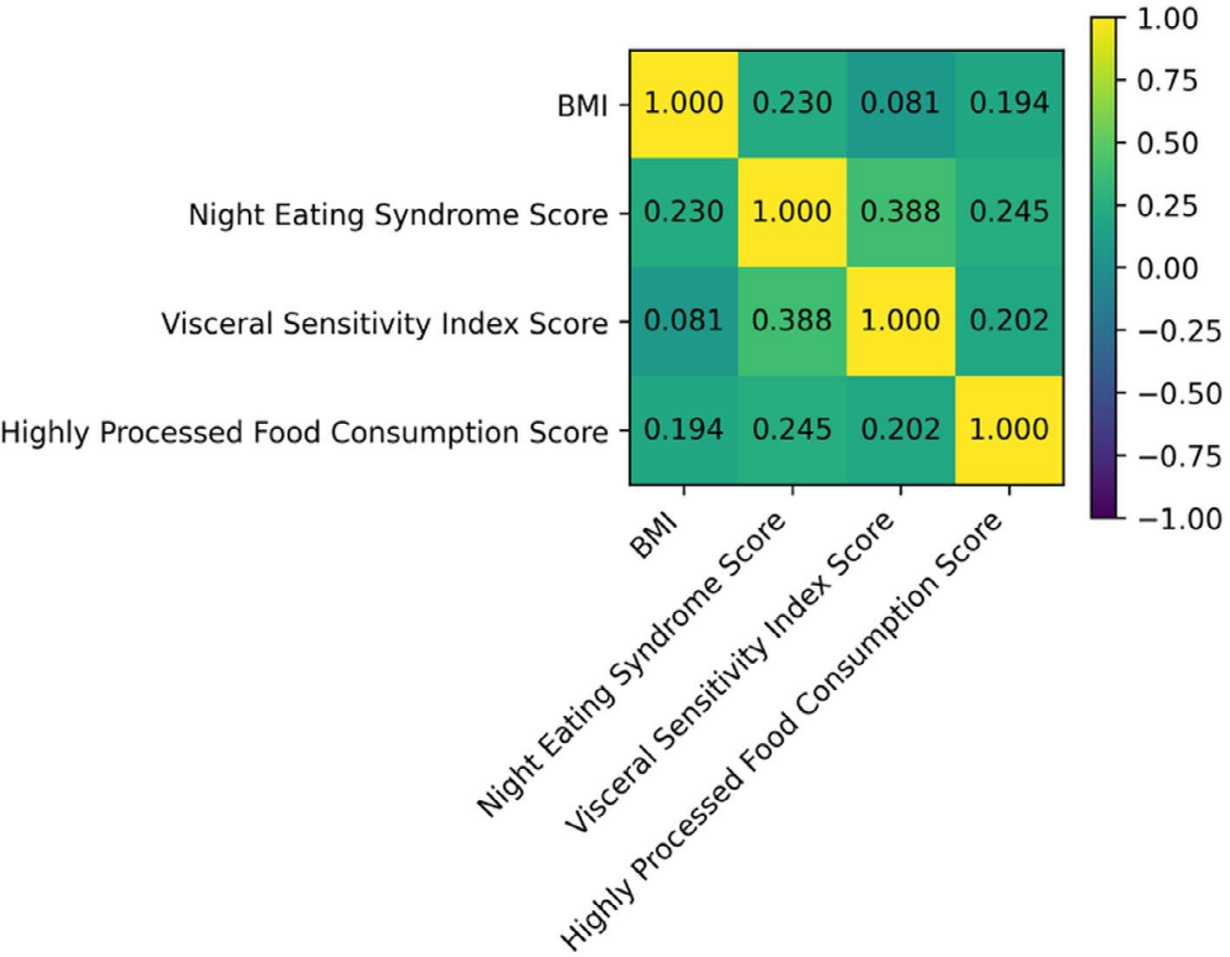
Correlation analysis for the prediction of highly processed food consumption, Night Eating Syndrome Questionnaire and Visceral Sensitivity Index total score, and BMI. BMI, body mass index.

Table 3 illustrates the associations between anthropometric and psychometric variables which are visually summarized in the correlation matrix heatmap (Figure 1). Weak but statistically significant positive correlations were found between BMI and both the NEQ and sQ‐HPF scores. A moderate positive relationship was observed between the severity of night eating (NEQ) and both GI‐specific anxiety (VSI) and UPF consumption. Furthermore, a weak positive correlation linked VSI scores with UPF intake.

**TABLE 3:** The relationship between ultra processed food consumption, Night Eating Syndrome Questionnaire score and Visceral Sensitivity Index Questionnaire score, and BMI.

	BMI	Night Eating Syndrome Questionnaire score	Visceral Sensitivity Index score	Screening Questionnaire of Highly Processed Food Consumption score
BMI	—	—	—	—
Night Eating Syndrome Questionnaire score	*r* = 0.230 *p* < 0.001*	—	—	—
Visceral Sensitivity Index score	*r* = 0.081 *p* = 0.250	*r* = 0.388 *p* < 0.001*	—	—
Screening Questionnaire of Highly Processed Food Consumption score	*r* = 0.194 *p* = 0.005*	*r* = 0.245 *p* < 0.001*	*r* = 0.202 *p* = 0.004*	—

*Note:* *r* represents the Pearson correlation coefficient.

Abbreviation: BMI, body mass index.

* Statistically significant at the *p* < 0.05.

When comparing groups based on diet quality (Table 4), women with high UPF consumption exhibited significantly higher NEQ scores and a greater prevalence of obesity compared to the low‐consumption group. High UPF intake was also associated with more frequent eating out and higher consumption of sugar‐rich foods. These primary associations were confirmed by non‐parametric sensitivity analyses, where the correlation between visceral sensitivity and NEQ scores remained highly significant (Spearman's rho = 0.388, *p* < 0.001).

**TABLE 4:** Evaluation of individuals' characteristics according to ultra processed food (UPF) consumption levels (sQ‐HPF score).

Variables	Ultra processed food consumption		*p*
	Low consumption (*n* = 134)	High consumption (*n* = 71)	
Age (years)	25.76 ± 3.66	25.96 ± 4.05	0.673
Visceral Sensitivity Index total score	36.95 ± 14.95	43.33 ± 16.6	0.100
Night Eating Syndrome Questionnaire total score	16.77 ± 8.31	20.54 ± 9.41	0.001*
Screening Questionnaire of Highly Processed Food Consumption total score	3.90 ± 1.21	7.79 ± 1.39	< 0.001*
BMI	25.64 ± 5.4	27.69 ± 6.94	0.056
Income status			
Less than expenses	39 (29.1)	19 (26.8)	0.184
Equal to expenses	20 (14.9)	18 (25.4)	
More than expenses	75 (56.0)	34 (47.9)	
Smoking			
Yes	39 (29.1)	11 (15.5)	0.031
No	95 (70.9)	60 (84.5)	
Alcohol consumption			
Yes	40 (29.9)	12 (16.9)	0.043
No	94 (70.1)	59 (83.1)	
Symptoms of polycystic ovarian syndrome			
Menstrual irregularity	105 (63.6)	60 (36.4)	0.291
Hirsutism	105 (63.6)	60 (36.4)	0.291
Acne	91 (65.9)	47 (34.1)	0.803
Infertility	17 (85.0)	3 (15.0)	0.052
Obesity	57 (74.0)	20 (26.0)	0.043*
Hair Loss	89 (66.9)	44 (33.1)	0.526
Fatigue	125 (66.1)	64 (33.9)	0.425
BMI classification			
Underweight	4 (3.0)	3 (4.2)	0.106
Normal	53 (39.6)	34 (47.9)	
Overweight	35 (26.1)	23 (32.4)	
Obese	42 (31.3)	11 (15.5)	
Frequency of eating out of the house			
None	2 (1.5)	3 (4.2)	0.031*
Every day	5 (3.7)	1 (1.4)	
Once or twice a week	60 (44.8)	21 (29.6)	
Three or four times a week	23 (17.2)	7 (9.9)	
Five or six times a week	5 (3.7)	5 (7.0)	
Once or twice a month	39 (29.1)	34 (47.9)	
Frequency of consumption of sugar‐containing foods			
None	0 (0.0)	2 (2.8)	< 0.001*
Every day	76 (56.7)	21 (29.6)	
Once or twice a week	14 (10.4)	25 (35.2)	
Three or four times a week	25 (18.7)	17 (23.9)	
Five or six times a week	17 (12.7)	2 (2.8)	
Once or twice a month	2 (1.5)	4 (5.6)	
Night eating syndrome			
Yes	33 (24.6)	10 (14.1)	0.078
No	101 (75.4)	61 (85.9)	

*Note:* Independent samples *t*‐test was used for comparing continuous variables between groups, and Chi‐square test was employed for categorical variables.

Abbreviation: BMI, body mass index.

* Statistically significant at the *p* < 0.05.

Table 5 presents the evaluation of participants' general characteristics according to their NES symptoms status. Women categorized with the severity of NES (NEQ score ≥ 25) exhibited significantly higher GI symptom‐specific anxiety (VSI) total scores, UPF consumption (sQ‐HPF scores), and current BMI values compared to those without NES (*p* < 0.05). Furthermore, individuals in the NES group showed higher rates of smoking and alcohol consumption (*p* < 0.05). Regarding PCOS‐related clinical features, infertility and a history of obesity since the time of PCOS diagnosis were significantly more prevalent among women with the severity of NES (*p* < 0.05).

**TABLE 5:** Evaluation of individuals' characteristics according to night eating syndrome.

Variables	Night eating syndrome		*p*
	No (*n* = 162)	Yes (*n* = 43)	
Age (years)	25.70 ± 3.76	26.60 ± 4.41	0.176
Visceral Sensitivity Index total score	38.41 ± 15.72	51.33 ± 14.36	< 0.001*
Night Eating Syndrome Questionnaire total score	15.16 ± 4.14	34.60 ± 6.30	< 0.001*
Screening Questionnaire of Highly Processed Food Consumption total score	6.23 ± 2.28	7.23 ± 2.13	0.019*
BMI	26.07 ± 5.72	30.39 ± 8.07	< 0.001*
Smoking			
Yes	28 (17.3)	22 (51.2)	< 0.001*
No	34 (82.7)	21 (48.8)	
Alcohol consumption			
Yes	33 (20.4)	19 (44.2)	0.001*
No	129 (79.6)	24 (55.8)	
Symptoms of polycystic ovarian syndrome			
Menstrual Irregularity	128 (77.6)	37 (22.4)	0.072
Hirsutism	126 (76.4)	39 (23.6)	0.057
Acne	109 (79.0)	29 (21.0)	0.984
Infertility	11 (55.0)	9 (45.0)	0.005*
Obesity	50 (64.9)	27 (35.1)	< 0.001*
Hair Loss	102 (76.7)	31 (23.3)	0.265
Fatigue	148 (78.3)	41 (21.7)	0.386
BMI classification			
Underweight	7 (4.3)	0 (0.0)	0.001*
Normal	71 (43.8)	16 (37.2)	
Overweight	52 (32.1)	6 (14.0)	
Obez	32 (19.8)	21 (48.8)	
Ultra processed food consumption			
High Consumption	101 (62.3)	33 (76.7)	0.078
Low Consumption	61 (37.7)	10 (23.3)	

*Note:* Independent samples *t*‐test was used for comparing continuous variables between groups, and Chi‐square test was employed for categorical variables.

Abbreviation: BMI, body mass index.

* Statistically significant at the *p* < 0.05.

To identify independent predictors and account for potential residual confounding, multiple linear regression models were constructed. The models were adjusted for BMI, smoking status, alcohol consumption, marital status, and income levels (Table 6).

**TABLE 6:** Linear regression model for NEQ total score and VSI total score.

Independent variables	Model 1: NEQ				
	*β*	*t*	*p*	95% CI	VIF
VSI total score	0.337	5.757	< **0.001** *	0.222, 0.452	1.24
sQ‐HPF total score	0.096	1.599	0.111	−0.022, 0.214	1.18
BMI (kg/m^2^)	0.201	3.428	**0.001** *	0.086, 0.316	1.35
Smoking status	0.286	4.562	< **0.001** *	0.163, 0.409	1.12
Alcohol consumption	0.013	0.213	0.831	−0.107, 0.133	1.08
Income status	−0.187	−2.800	**0.006** *	−0.318, −0.056	1.15
Marital status	−0.123	−2.092	**0.038** *	−0.238, −0.008	1.21
Model statistics	*R* ^2^ = 0.390, *p* < 0.001				

	Model 2: VSI				
	*β*	*t*	*p*	95% CI	VIF
NEQ total score	0.430	5.757	< **0.001** *	0.284, 0.576	1.42
sQ‐HPF total score	0.124	1.844	0.067	−0.008, 0.256	1.15
BMI (kg/m^2^)	−0.061	−0.892	0.373	−0.195, 0.073	1.28
Smoking status	−0.093	−1.252	0.212	−0.239, 0.053	1.10
Alcohol consumption	0.014	0.195	0.846	−0.127, 0.155	1.06
Income status	0.113	1.467	0.144	−0.038, 0.264	1.12
Marital status	−0.028	−0.415	0.678	−0.160, 0.104	1.18
Model statistics	*R* ^2^ = 0.222, *p* < 0.001				

*Note:* Beta represents the standardized regression coefficient. Model 1: Dependent variable is the Night Eating Questionnaire (NEQ) total score. Model 2: Dependent variable is the Visceral Sensitivity Index (VSI) total score. Bold values in Table 6 represent statistically significant independent predictors (*p* < 0.05) with an asterisk (*). Additionally, the bold text under 'Model Statistics' highlights the overall model fit (R^2^) and its corresponding statistical significance (*p* < 0.001) for both Model 1 and Model 2.

Abbreviations: BMI, body mass index; NEQ, Night Eating Questionnaire; sQ‐HPF, Screening Questionnaire of Highly Processed Food Consumption; VSI, Visceral Sensitivity Index (GI‐specific anxiety).

* Significant at *p* < 0.05.

**FIGURE 2:**
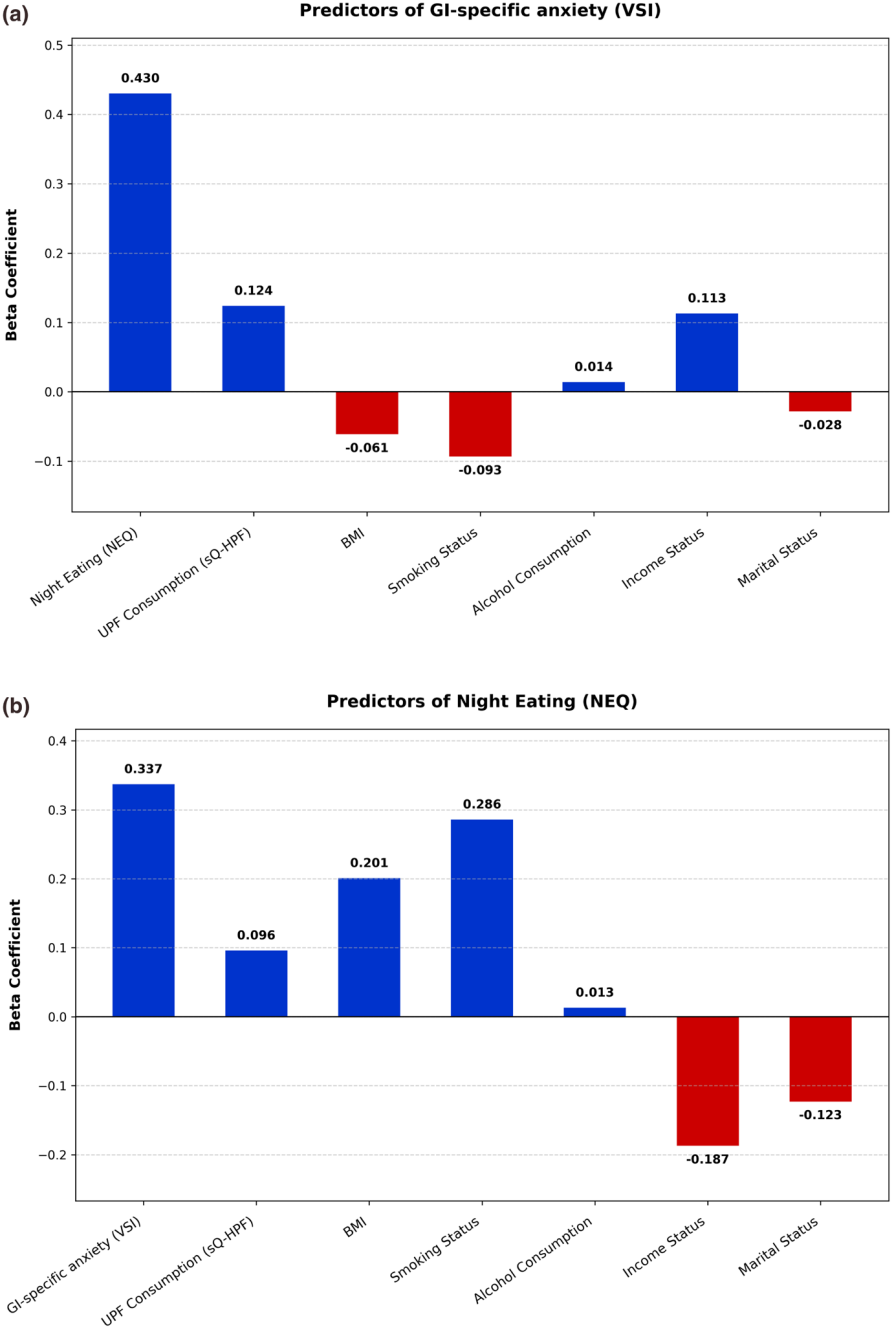
Standardized beta coefficients from multiple linear regression models. (a) Predictors of night eating severity (NEQ score): The model shows that GI‐specific anxiety (VSI), smoking status, and BMI are significant positive predictors, while income and marital status are negative predictors (*R* ^2^ = 0.390, *p* < 0.001). (b) Predictors of gastrointestinal (GI) symptom‐specific anxiety (VSI score): Night eating severity (NEQ) emerges as the primary significant independent predictor (*R* ^2^ = 0.222, *p* < 0.001). Blue bars indicate positive associations, while red bars indicate negative associations. All models were adjusted for BMI, smoking, alcohol use, and socioeconomic status. (a) Linear Regression Analysis for the Prediction of Visceral Sensitivity Index total score. (b) Linear Regression Analysis for the Prediction of Night Eating Syndrome Questionnaire total score.

In Model 1, where NEQ total score was the dependent variable, VSI total score (beta = 0.337, *t* = 5.757, *p* < 0.001) and smoking status (beta = 0.286, *t* = 4.562, *p* < 0.001) were identified as significant independent predictors, while BMI (beta = 0.201, *t* = 3.428, *p* = 0.001) and income status (beta = −0.187, *t* = −2.800, *p* = 0.006) emerged as modest independent predictors (*R* ^2^ = 0.390, *p* < 0.001; Figure 2a).

In Model 2, with GI symptom‐specific anxiety (VSI) as the dependent variable, the severity of night eating (NEQ scores) emerged as the sole significant independent predictor (*beta* = 0.430, *t* = 5.757, *p* < 0.001), even after adjusting for BMI, smoking status, and socioeconomic factors (*R* ^2^ = 0.222, *p* < 0.001). These findings indicate a robust and independent association between night eating symptoms and GI‐specific anxiety in women with PCOS (Figure 2b).

**FIGURE 3:**
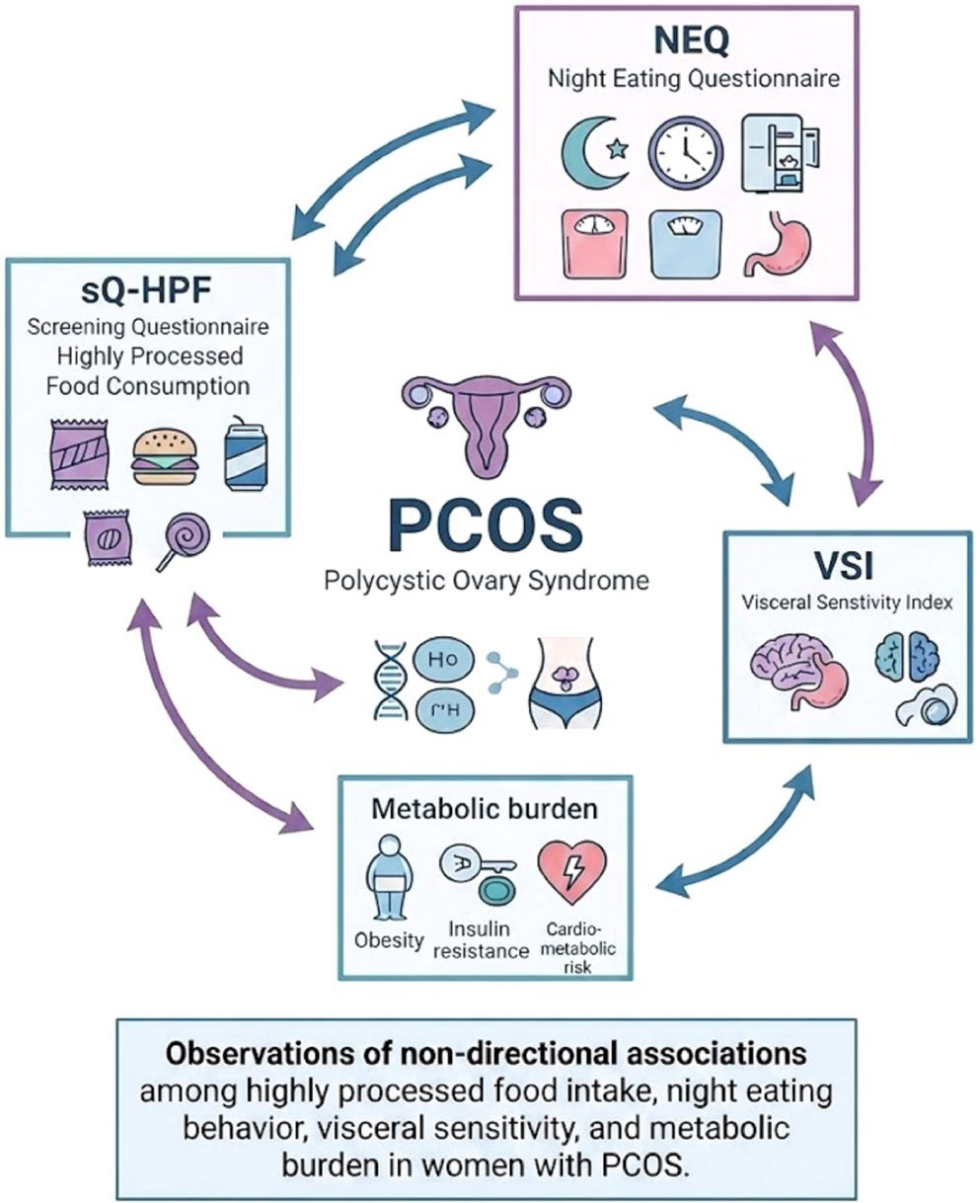
Conceptual diagram of the observed associations between ultra processed food consumption, night eating symptoms, and gastrointestinal‐specific anxiety in women with PCOS. The diagram illustrates the multidimensional interplay within the diet‐brain‐gut axis. Bidirectional arrows represent statistically significant associations, emphasizing a reciprocal relationship rather than causal precedence, consistent with the study's cross‐sectional design.

To synthesize these findings within the clinical context of PCOS, the observed non‐directional associations among highly processed food intake (sQ‐HPF), night eating behavior (NEQ), GI symptom‐specific anxiety (VSI), and underlying metabolic burden are conceptually integrated and illustrated in Figure 3.

The sensitivity analysis confirmed that the primary associations remained significant after excluding participants with BMI ≥ 35 kg/m^2^. Notably, the correlation between the severity of night eating (NEQ) and GI symptom‐specific anxiety (VSI) remained moderately strong and robust (*r* = 0.406, *p* < 0.001), as detailed in Table S1. Furthermore, to assess the robustness of our regression models, we performed a sensitivity analysis by excluding BMI from the multivariate models; the associations remained stable and significant, as presented in Table S2.

## 4 Discussion

PCOS is one of the most common endocrine disorders in women of reproductive age and is associated with metabolic, hormonal, and psychological symptoms (The Thessaloniki ESHRE/ASRM‐Sponsored PCOS Consensus Workshop Group 2008). Factors such as nutritional habits, eating behaviors, and GI health are becoming increasingly important in the management of PCOS (Teede et al. 2023). Current evidence indicates that UPF consumption is associated with obesity, insulin resistance, and inflammation, and that night eating symptoms are more common in women with PCOS (Ozgen Saydam and Yildiz 2021; Shoaibinobarian et al. 2022). However, to our knowledge, no previous study has simultaneously examined the relationships between UPF consumption, night eating, and visceral sensitivity (specifically GI symptom‐specific anxiety) in women with PCOS. In this study, we evaluated these associations and observed several significant relationships. Briefly, (i) more than half of the women with PCOS were overweight or obese; (ii) approximately one‐third reported a high level of UPF consumption, and one‐fifth met the criteria for NES; (iii) high UPF consumption was accompanied by higher NEQ scores, more frequent obesity, higher sugary food intake, and more frequent eating outside the home; (iv) women categorized with NES had higher BMI, sQ‐HPF scores, and GI‐specific anxiety scores, alongside higher rates of smoking, alcohol use, and a higher prevalence of infertility and obesity symptoms; (v) GI‐specific anxiety was positively correlated with sQ‐HPF scores; and (vi) in regression analyses, GI‐specific anxiety and BMI were identified as independent predictors of NEQ scores, while NEQ scores emerged as the primary significant predictor of GI‐specific anxiety. A significant strength of the current study is that the independent association between GI‐specific anxiety (VSI scores) and the severity of night eating (NEQ scores) remained robust even after adjusting for multiple confounders, including BMI, smoking status, and socioeconomic factors. This relationship suggests that the potential interplay between GI‐related distress and maladaptive eating patterns in women with PCOS may not be merely a reflection of general lifestyle factors or obesity, but rather a specific behavioral‐cognitive association that warrants clinical attention.

Our findings showed that more than half of the women with PCOS were overweight or obese. This result is consistent with previous studies reporting high rates of overweight and obesity in this population (Bykowska‐Derda et al. 2021; Van Hooff et al. 1999). In a cohort of 65,665 women followed for 24 years, the mean duration since PCOS diagnosis was 28 years and women with PCOS were more likely to be overweight or obese both in childhood and adulthood (Aarestrup et al. 2021). Although biochemical markers were not assessed in the present study, insulin resistance is widely considered to play a bidirectional role in the relationship between PCOS and obesity, contributing both to PCOS pathogenesis and emerging as a consequence of obesity. In women with PCOS, the risk of impaired glucose tolerance has been reported to be three times higher than in healthy women, independent of BMI (Kakoly et al. 2018). Theoretically, insulin resistance and compensatory hyperinsulinemia may exacerbate hyperandrogenaemia by stimulating androgen production and decreasing sex hormone binding globulin synthesis (Winters et al. 2014). It is also hypothesized that hyperandrogenism, may promote insulin resistance and obesity by increasing abdominal fat and oxidative stress (Dumesic et al. 2020). The relationship between PCOS and increased obesity has also been linked to disturbed eating behaviors and eating disorders. Symptoms such as hirsutism, infertility and obesity may impair body image, reduce quality of life and increase depression risk, potentially predisposing women with PCOS to disordered eating (Hall et al. 2019; Krug et al. 2019). A high prevalence of disordered eating and eating disorders has been reported in women with PCOS, and these factors have been shown to contribute to higher body weight (Asdaq et al. 2020). In line with these proposed mechanisms, lifestyle intervention is considered the first strategy to improve the metabolic and hormonal profile in overweight or obese women with PCOS, and a meta‐analysis has shown that lifestyle interventions including nutritional therapy can improve free androgen levels and BMI in this population (Lim et al. 2019).

The present study also examined the prevalence of UPF consumption and the severity of night eating symptoms in women with PCOS, contributing to the literature that emphasizes the importance of assessing dietary habits for optimal management. We found that 34.6% of participants were categorized into the high UPF consumption group based on their sQ‐HPF scores. Ultra‐processed food intake has been increasing globally, and in some populations more than half of daily energy intake is derived from such products (Krug et al. 2019). These foods typically have high energy density, and excessive consumption is considered a significant factor that may contribute to weight gain and obesity (Hall et al. 2019). In addition, various additives used in ultra‐processed foods have been suggested to promote oxidative stress, inflammation, and endothelial dysfunction (Poursalehi et al. 2024). Although biological markers were not evaluated in our study, literature suggests that high ultra‐processed food consumption is often associated with alterations in gut microbiota composition, reduced short‐chain fatty acid production, dysbiosis, and inflammation. Furthermore, it is hypothesized that reduced GLP‐1 and PYY levels and increased ghrelin concentrations might be observed with high ultra‐processed food intake, which could unfavorably influence appetite regulation and eating behavior (Song et al. 2023). Artificial sweeteners and monosodium glutamate contained in some ultra‐processed foods have been theoretically linked to changes in neurotransmitters such as norepinephrine and serotonin, potentially contributing to depressive symptoms (Lane et al. 2022). Taken together, proposed mechanisms support a potential hypothetical link between ultra‐processed food consumption and PCOS. Although women with PCOS frequently exhibit unhealthy eating habits (Shishehgar et al. 2016), and high ultra‐processed food consumption has been associated with increased PCOS risk and craving for ultra‐processed foods (Shoaibinobarian et al. 2022), studies directly investigating ultra‐processed food intake in women with PCOS remain limited. For example, in a study of 122 women with PCOS, 30% frequently consumed ultra‐processed fast foods outside the home (Bykowska‐Derda et al. 2021). In another study conducted in Türkiye, it was found that packaged product consumption was higher in women with PCOS compared to controls, which subsequently reduced overall diet quality (Akçakaya 2023). Furthermore, insulin resistance, which is common in PCOS, may potentially alter the brain's reward mechanisms and appetite‐related hormones, possibly increasing the consumption of foods rich in refined carbohydrates, sugar, and fat (Al Massadi et al. 2019; Hajivandi et al. 2020). This could lead to a tendency toward ultra‐processed foods and, consequently, contribute to eating disorders such as night eating syndrome. Impaired eating behaviors might thus favor ultra‐processed food intake, further aggravating insulin resistance, obesity, and PCOS symptoms (Ayton et al. 2021). Our findings that women with high UPF consumption exhibited higher NEQ scores and a higher prevalence of obesity align with these theoretically proposed mechanisms.

Symptoms such as hirsutism, acne, and infertility in women with PCOS are often associated with lower self‐esteem, higher anxiety, and increased risk of depression due to their inconsistency with social beauty standards and gender norms. These psychosocial difficulties may potentially promote emotional eating, disturbed eating patterns, and eating disorders. Body dissatisfaction due to obesity, repeated unsuccessful dieting attempts, and overly restrictive diets may further increase the risk of disordered eating (Kogure et al. 2019). Another theoretically proposed mechanism involves the potential effects of hyperandrogenism on appetite and impulse control; it is hypothesized that high testosterone and low estrogen levels may adversely affect satiety signaling and increase vulnerability to emotional hunger. While not directly assessed in our study, the development of insulin resistance may also contribute to hyperandrogenism, potentially elevating cortisol levels and disrupting neurotransmitter release, thereby amplifying these mechanisms (Lalonde‐Bester et al. 2024). Despite the increased risk of eating disorders in women with PCOS, the number of studies focusing specifically on NES is limited. One study reported that 12.9% of women with PCOS had NES (Lee et al. 2017), whereas prevalence estimates in the general population range between 1.5% and 5.7% and are related to BMI classification (De Zwaan et al. 2014). In Türkiye, the prevalence of NES in the general population has been reported as 10%, with a significant association between obesity and night eating (Sutcu et al. 2021). In our study, the prevalence of the severity of NES was 21% in women with PCOS, higher than these general population estimates and some studies in PCOS samples. Moreover, the severity of night eating symptoms was higher in the high UPF consumption group, a finding supported by our bivariate analyses and group comparisons. Considering the high prevalence of obesity in women with PCOS, alongside the challenges in maintaining recommended lifestyle modifications, screening for UPF consumption and night eating symptoms may be highly beneficial in clinical practice.

Our results also indicated that night eating symptoms in women with PCOS were associated not only with sQ‐HPF scores but also with BMI, GI symptom‐specific anxiety (VSI scores), PCOS symptoms, and lifestyle factors including smoking and alcohol consumption (Table 5). Contrary to a study reporting no significant association between BMI and night eating severity in women with PCOS (Yüksel et al. 2024), our findings showed higher BMI in those categorized with NES, which aligns with literature demonstrating associations between disturbed eating patterns and BMI (Greenwood et al. 2020; Jeanes et al. 2017; Moghetti and Tosi 2021; Stefanaki et al. 2023). Associations between disordered eating and PCOS symptoms such as obesity, acne, hirsutism, and depression have been previously reported (Çoban et al. 2019; Greenwood et al. 2020; Iwasa et al. 2018; Stefanaki et al. 2023). Consistent with this, we found that infertility and obesity symptoms were more common among women with NES. Although some studies have found no significant relationship between smoking and night eating syndrome in women with PCOS (Greenwood et al. 2020; Yang et al. 2024; Yüksel et al. 2024), our results indicated that smoking and alcohol consumption were more frequent in those with NES. These findings suggest that night eating symptoms in PCOS may be part of a broader clinical and psychosocial risk profile that includes substance use and core clinical symptoms. The inclusion of lifestyle factors in our adjusted regression model revealed smoking status as a prominent predictor of night eating scores. This finding is consistent with literature suggesting that nicotine dependence often co‐occurs with circadian rhythm disruptions and disordered eating patterns. Furthermore, the observation that the independent effect of UPF consumption was attenuated in the multivariate model (*p* = 0.111) suggests that highly processed food intake may cluster with other risk factors, such as smoking and psychological distress, collectively contributing to the metabolic and clinical burden in PCOS.

Regarding potential biological pathways, literature suggests that gut microbiota and dysbiosis may play a role in hyperandrogenism. The intestinal microbiota is hypothesized to be involved in bile acid metabolism, and it has been proposed that dysbiosis‐related alterations in circulating bile acids could potentially be linked to hyperandrogenism in women with PCOS (Derrigo and LaFata 2023). These risks appear to be higher in the presence of obesity accompanying PCOS (Allison et al. 2008). While not directly measured in the current sample, dysbiosis might also theoretically influence the perception of GI discomfort by promoting hyperandrogenism, adversely affecting neuromuscular function through the brain‐gut axis, and inducing low‐grade chronic inflammation (Lupu et al. 2023). In this context, visceral sensitivity (as measured by the VSI) encompasses not only GI symptoms and pain but also the cognitive‐affective dimension, including fears and anxiety related to symptom occurrence. It has been associated with poor GI health, visceral pain, emotional dysregulation, depression and anxiety (Zvolensky et al. 2018). It is plausible that pain‐related mechanisms could be influenced by sex‐related neurological and hormonal differences; for instance, theoretical models suggest that endogenous estrogen levels may potentially affect pain sensitivity (Vincent et al. 2013). Animal studies have also suggested that visceral sensitivity may be potentially linked to estrogen effects (Pujo et al. 2023). To our knowledge, there is no previous study that has specifically investigated GI‐specific anxiety (VSI scores) in women with PCOS or their relationship with eating behaviors. It has been hypothesized that while androgens may have a protective role in regulating visceral pain and inflammation by reducing proinflammatory components in conditions such as IBS, and that this potential mechanism may paradoxically increase visceral pain and symptoms in women with PCOS (Mulak et al. 2014). In the present study, we observed positive correlations between GI symptom‐specific anxiety (VSI scores), UPF consumption, and the severity of night eating. In regression analyses, night eating symptoms emerged as a significant predictor of GI‐specific anxiety, whereas the independent association with UPF consumption did not reach statistical significance. Although no previous study has simultaneously examined GI‐specific anxiety and NES symptoms in PCOS, Cherlin et al. (2024) reported that the frequency of visceral pain in women with PCOS was 16.75%, increasing to 42.68% in the presence of comorbid mental disorders. Furthermore, an intervention study involving women with PCOS and severe visceral pain reported reductions in symptoms following a program that included exercise and the restriction of UPF and night eating (Shrivastava et al. 2024). Consistent with these findings, we observed higher VSI scores in women with PCOS categorized with NES compared to those without, suggesting that night eating patterns are closely linked to the psychological burden of GI symptoms. Regarding the construct validity of our measures, it is essential to interpret VSI scores specifically as a measure of GI symptom‐specific anxiety. The VSI is designed to capture cognitive‐affective responses, such as hypervigilance and fear of GI symptoms, rather than direct physiological nociception. Our results suggest that this psychological burden is a key factor associated with night eating, potentially mediated through stress‐induced eating mechanisms where individuals consume energy‐dense foods to cope with GI‐related anxiety.

This study has several strengths. First, it provides a holistic perspective by simultaneously examining UPF consumption, night eating, and GI symptom‐specific anxiety (VSI) in women with PCOS. To our knowledge, no previous study has evaluated these three factors together in this population, helping to fill a literature gap and highlighting the importance of considering both nutritional patterns and night eating symptoms in PCOS management. Second, the instruments used (specifically the sQ‐HPF, NEQ, and VSI) are validated for the Turkish population. Notably, in the present study, these scales demonstrated strong internal consistency (Cronbach's alpha = 0.61, 0.80, and 0.95, respectively), further increasing the reliability of our measurements. It should be noted that while NEQ and VSI were analyzed in separate models, a conceptual overlap exists between night eating and GI‐specific anxiety. These constructs likely influence each other in a bidirectional manner; however, our study highlights UPF consumption as a consistent associate of both, suggesting a broad impact of diet quality on the diet‐brain‐gut axis in PCOS.

Nevertheless, some limitations of the present study warrant consideration. First and foremost, the cross‐sectional design of this study precludes the establishment of temporal precedence or causal relationships between UPF consumption, night eating, and GI‐specific anxiety. Longitudinal studies are required to determine whether maladaptive eating patterns precede or follow GI distress in PCOS. Second, all data, including dietary intake, anthropometric measurements, and psychometric scores, were collected via self‐report measures, which may introduce recall bias or social desirability bias. Although anthropometric measurements were obtained via self‐report, which may introduce misclassification bias (specifically the potential under‐reporting of weight in higher BMI categories), our sensitivity analysis (excluding BMI from multivariate models and performing subgroup analyses for BMI < 35 kg/m^2^) demonstrated that the associations remained robust (as detailed in Table S2). This suggests that the clinical‐psychological interplay observed is stable regardless of potential BMI measurement error.

Third, while the supervised clinical setting ensured a high completion rate (> 95%) and minimized non‐response bias, the lack of stratification by PCOS phenotypes (A‐D) may introduce residual confounding. However, the high prevalence of hyperandrogenism and menstrual irregularities in our sample (80.5%) suggests that it adequately reflects common clinical presentations of the syndrome. Furthermore, the explanatory power of our regression models (*R* ^2^ values of 0.390 and 0.222) indicates that a substantial proportion of the variance in night eating and GI‐specific anxiety remains unexplained. This is a common finding in behavioral nutrition research, reflecting the multifactorial nature of PCOS‐related symptoms. The residual variance may be attributed to unmeasured factors, such as circadian rhythm disruptions, hormonal fluctuations (e.g., cortisol or ghrelin levels), and gut microbiota diversity, as well as the absence of standardized assessments for physical activity levels and total daily energy intake. Additionally, while we focused on GI‐specific anxiety, the lack of clinical scales for generalized psychological distress (e.g., depression) remains a limitation that should be addressed in future predictive models to enhance explanatory power. The NEQ demonstrated strong internal consistency in our sample (Cronbach's alpha = 0.80), as did the VSI (alpha = 0.95). It should be considered that UPF consumption in this study was assessed using a screening tool rather than a quantitative dietary assessment method. Therefore, the findings reflect relative consumption patterns rather than precise dietary intake, and potential misclassification may have influenced the observed associations. Finally, participants were recruited from a specific clinical setting, which may introduce selection bias; however, this approach was justified by the necessity of ensuring high diagnostic accuracy through physician‐confirmed PCOS diagnoses, a level of rigor often absent in general community‐based samples.

Furthermore, the VSI specifically captures the cognitive‐affective dimension of GI symptom‐specific anxiety, such as hypervigilance and fear, rather than direct physiological nociception; thus, findings must be interpreted strictly within this psychometric framework. Additionally, women from rural areas were under‐represented in our sample, which may limit the generalizability of the findings to the broader PCOS population. Future studies utilizing food diaries, repeated 24‐h dietary recalls, and biochemical markers would provide a more objective assessment of UPF intake. Despite these limitations, this study provides an original and holistic contribution by underlining the necessity of a comprehensive assessment of nutrition, night eating symptoms, and GI‐specific anxiety in the clinical management of PCOS.

## 5 Conclusion

In conclusion, this study demonstrates a significant association between UPF consumption, night eating symptoms, and GI‐specific anxiety in women with PCOS. Our findings suggest a complex interplay where poor diet quality and maladaptive eating patterns are linked to increased psychological distress related to GI symptoms. Specifically, the independent relationship between night eating and GI‐specific anxiety remains robust even after adjusting for metabolic and lifestyle factors. These results emphasize the importance of a multidisciplinary approach in PCOS management, highlighting that screening for dietary habits and behavioral eating patterns is associated with a more comprehensive understanding of the patient's clinical profile. Future longitudinal research is necessary to clarify the directional nature of these associations and to determine whether dietary interventions can alleviate the psychological burden of GI symptoms in this population.

These results highlight that nutritional interventions in women with PCOS should move beyond a narrow focus on weight control and also target diet quality and eating patterns. Limiting UPF consumption, implementing multidisciplinary strategies to regulate night eating symptoms, and adopting nutritional approaches that support GI health may be vital components of a holistic management plan for PCOS. Given the complex interplay between metabolic, GI, and psychological factors in this population, integrated care that combines dietary counseling, behavioral‐cognitive interventions, and the management of GI symptoms may offer a more effective strategy for improving long‐term outcomes. Further longitudinal and interventional studies are warranted to confirm these associations and to develop targeted treatment approaches aimed at optimizing both the metabolic and reproductive health of women with PCOS.

## Author Contributions

**Emine Merve Ekici:** conceptualization, investigation, funding acquisition, writing – original draft, methodology, validation, visualization, writing – review and editing, software, formal analysis, project administration, data curation, supervision, resources. **Vefa Şakar:** methodology, validation, project administration, data curation, conceptualization, investigation, funding acquisition, writing – original draft. **Özge Mengi Çelik:** conceptualization, investigation, funding acquisition, writing – original draft, methodology, validation, visualization, writing – review and editing, software. **Sena Dilşad Akçakaya:** investigation, funding acquisition, writing – original draft, conceptualization, methodology, validation, visualization, writing – review and editing, software, project administration, data curation, resources.

## Funding

The authors have nothing to report.

## Ethics Statement

All procedures performed in studies involving human participants were in accordance with the ethical standards of the institutional research committee and with the 1964 Helsinki declaration and its later amendments or comparable ethical standards. The study was conducted with the approval of the University of Health Sciences Gulhane Scientific Research Ethics Committee, numbered 2024/547.

## Consent

The authors have nothing to report. Consent to participate: Informed consent was obtained from all participants included in the study.

## Conflicts of Interest

The authors declare no conflicts of interest.

## Supporting information


**Table S1:** Correlations among BMI, night eating, GI symptom‐specific anxiety, and ultra‐processed food consumption in women with PCOS.
**Table S2:** Sensitivity analysis for GI symptom‐specific anxiety excluding BMI.
**Appendix S1:** Participant informed consent form.

## Data Availability

The data that support the findings of this study are available from the corresponding author upon reasonable request.

## References

[fsn372007-bib-0001] Aarestrup, J. , D. C. Pedersen , P. E. Thomas , et al. 2021. “Birthweight, Childhood Body Mass Index, Height and Growth, and Risk of Polycystic Ovary Syndrome.” Obesity Facts 14, no. 3: 283–290. 10.1159/000515294. 33979806 PMC8255636

[fsn372007-bib-0002] Abay, B. 2024. İrritabl bağırsak sendromlu hastalarda gastrointestinal semptom derecelendirme ölçeği‐İrritabl Bağırsak Sendromu (İBS) versiyonu ve Visseral Duyarlılık İndeksinin geçerlik ve güvenirlik çalışması [Yüksek Lisans Tezi]. Zonguldak Bülent Ecevit Üniversitesi.

[fsn372007-bib-0003] Akçakaya, S. D. 2023. “Polikistik Over sendromlu kadınlarda yaşam kalitesi ve akdeniz diyetine uyumun değerlendirilmesi [Yüksek Lisans Tezi, Lokman Hekim Üniversitesi].” Ulusal Tez Merkezi (YÖK Tez). https://tez.yok.gov.tr/UlusalTezMerkezi/TezGoster?key=S2eMu1TIwY_v4mYv58xArz7YxwQ1fd7ywrMaivZW6Zc4y18tX_NCFt8QDKtA3if7.

[fsn372007-bib-0004] Al Massadi, O. , R. Nogueiras , C. Dieguez , and J.‐A. Girault . 2019. “Ghrelin and Food Reward.” Neuropharmacology 148: 131–138. 10.1016/j.neuropharm.2019.01.001. 30615902

[fsn372007-bib-0005] Allison, K. C. , J. D. Lundgren , J. P. O'Reardon , et al. 2008. “The Night Eating Questionnaire (NEQ): Psychometric Properties of a Measure of Severity of the Night Eating Syndrome.” Eating Behaviors 9, no. 1: 62–72. 10.1016/j.eatbeh.2007.03.007. 18167324

[fsn372007-bib-0006] Asdaq, S. M. B. , S. Jomah , R. Hasan , et al. 2020. “Impact of Polycystic Ovary Syndrome on Eating Behavior, Depression and Health Related Quality of Life: A Cross‐Sectional Study in Riyadh.” Saudi Journal of Biological Sciences 27, no. 12: 3342–3347. 10.1016/j.sjbs.2020.08.039. 33304140 PMC7715018

[fsn372007-bib-0007] Askari, M. , J. Heshmati , H. Shahinfar , N. Tripathi , and E. Daneshzad . 2020. “Ultra‐Processed Food and the Risk of Overweight and Obesity: A Systematic Review and Meta‐Analysis of Observational Studies.” International Journal of Obesity 44, no. 10: 2080–2091. 10.1038/s41366-020-00650-z. 32796919

[fsn372007-bib-0008] Atasoy, N. , O. Saracli , N. Konuk , et al. 2014. “The Reliability and Validity of Turkish Version of the Night Eating Questionnaire in Psychiatric Outpatient Population.” Anatolian Journal of Psychiatry 15, no. 3: 238. 10.5455/apd.39829.

[fsn372007-bib-0009] Ayton, A. , A. Ibrahim , J. Dugan , E. Galvin , and O. W. Wright . 2021. “Ultra‐Processed Foods and Binge Eating: A Retrospective Observational Study.” Nutrition 84: 111023. 10.1016/j.nut.2020.111023. 33153827

[fsn372007-bib-0010] Barrea, L. , A. Arnone , G. Annunziata , et al. 2019. “Adherence to the Mediterranean Diet, Dietary Patterns and Body Composition in Women With Polycystic Ovary Syndrome (PCOS).” Nutrients 11, no. 10: 2278. 10.3390/nu11102278. 31547562 PMC6836220

[fsn372007-bib-0011] Bykowska‐Derda, A. , M. Czlapka‐Matyasik , M. Kaluzna , M. Ruchala , and K. Ziemnicka . 2021. “Diet Quality Scores in Relation to Fatness and Nutritional Knowledge in Women With Polycystic Ovary Syndrome: Case–Control Study.” Public Health Nutrition 24, no. 11: 3389–3398. 10.1017/S1368980020001755. 32693854 PMC10195304

[fsn372007-bib-0012] Cherlin, T. , S. Mohammed , S. Ottey , K. Sherif , and S. S. Verma . 2024. “Understanding Pain in Polycystic Ovary Syndrome: Health Risks and Treatment Effectiveness.” Endocrinology (Including Diabetes Mellitus and Metabolic Disease). 10.1101/2024.10.15.24315513.

[fsn372007-bib-0013] Çoban, Ö. G. , Ö. D. Tulacı , A. S. Adanır , and A. Önder . 2019. “Psychiatric Disorders, Self‐Esteem, and Quality of Life in Adolescents With Polycystic Ovary Syndrome.” Journal of Pediatric and Adolescent Gynecology 32, no. 6: 600–604. 10.1016/j.jpag.2019.07.008. 31362114

[fsn372007-bib-0014] Davis, C. , C. Curtis , R. D. Levitan , J. C. Carter , A. S. Kaplan , and J. L. Kennedy . 2011. “Evidence That “Food Addiction” Is a Valid Phenotype of Obesity.” Appetite 57, no. 3: 711–717. 10.1016/j.appet.2011.08.017. 21907742

[fsn372007-bib-0015] De Zwaan, M. , A. Müller , K. C. Allison , E. Brähler , and A. Hilbert . 2014. “Prevalence and Correlates of Night Eating in the German General Population.” PLoS One 9, no. 5: e97667. 10.1371/journal.pone.0097667. 24828066 PMC4020826

[fsn372007-bib-0016] Derrigo, K. , and E. M. LaFata . 2023. “Examining the Proportions of Food Addiction Among Women With and Without Polycystic Ovarian Syndrome Who Do and Do Not Take Hormonal Birth Control.” Eating Behaviors 51: 101824. 10.1016/j.eatbeh.2023.101824. 37950975

[fsn372007-bib-0017] Dumesic, D. A. , D. H. Abbott , S. Sanchita , and G. D. Chazenbalk . 2020. “Endocrine–Metabolic Dysfunction in Polycystic Ovary Syndrome: An Evolutionary Perspective.” Current Opinion in Endocrine and Metabolic Research 12: 41–48. 10.1016/j.coemr.2020.02.013. 32363240 PMC7194185

[fsn372007-bib-0018] Erdoğan Gövez, N. , E. Köksal , C. Martinez‐Perez , and L. Daimiel . 2024. “Validity and Reliability of the Turkish Version of the Screening Questionnaire of Highly Processed Food Consumption (sQ‐HPF).” Nutrients 16, no. 15: 2552. 10.3390/nu16152552. 39125430 PMC11314031

[fsn372007-bib-0019] Gambineri, A. , C. Pelusi , V. Vicennati , U. Pagotto , and R. Pasquali . 2002. “Obesity and the Polycystic Ovary Syndrome.” International Journal of Obesity and Related Metabolic Disorders: Journal of the International Association for the Study of Obesity 26, no. 7: 883–896. 10.1038/sj.ijo.0801994. 12080440

[fsn372007-bib-0020] Garvey, W. T. , J. I. Mechanick , E. M. Brett , et al. 2016. “American Association Of Clinical Endocrinologists and American College of Endocrinology Comprehensive Clinical Practice Guidelines For Medical Care Of Patients With Obesity Executive Summary Complete Guidelines.” Endocrine Practice 22, no. 7: 842–884. 27472012 10.4158/EP161356.ESGL

[fsn372007-bib-0021] Greenwood, E. A. , L. A. Pasch , M. I. Cedars , and H. G. Huddleston . 2020. “Obesity and Depression Are Risk Factors for Future Eating Disorder‐Related Attitudes and Behaviors in Women With Polycystic Ovary Syndrome.” Fertility and Sterility 113, no. 5: 1039–1049. 10.1016/j.fertnstert.2020.01.016. 32386615

[fsn372007-bib-0022] Hajivandi, L. , M. Noroozi , F. Mostafavi , and M. Ekramzadeh . 2020. “Food Habits in Overweight and Obese Adolescent Girls With Polycystic Ovary Syndrome (PCOS): A Qualitative Study in Iran.” BMC Pediatrics 20, no. 1: 277. 10.1186/s12887-020-02173-y. 32498675 PMC7271429

[fsn372007-bib-0023] Hall, K. D. , A. Ayuketah , R. Brychta , et al. 2019. “Ultra‐Processed Diets Cause Excess Calorie Intake and Weight Gain: An Inpatient Randomized Controlled Trial of Ad Libitum Food Intake.” Cell Metabolism 30, no. 1: 67–77.e3. 10.1016/j.cmet.2019.05.008. 31105044 PMC7946062

[fsn372007-bib-0024] Iwasa, T. , T. Matsuzaki , K. Yano , et al. 2018. “Effects of Chronic Testosterone Administration on the Degree of Preference for a High‐Fat Diet and Body Weight in Gonadal‐Intact and Ovariectomized Female Rats.” Behavioural Brain Research 349: 102–108. 10.1016/j.bbr.2018.02.021. 29544963

[fsn372007-bib-0025] Jeanes, Y. M. , S. Reeves , E. L. Gibson , C. Piggott , V. A. May , and K. H. Hart . 2017. “Binge Eating Behaviours and Food Cravings in Women With Polycystic Ovary Syndrome.” Appetite 109: 24–32. 10.1016/j.appet.2016.11.010. 27825940

[fsn372007-bib-0026] Kakoly, N. S. , M. B. Khomami , A. E. Joham , et al. 2018. “Ethnicity, Obesity and the Prevalence of Impaired Glucose Tolerance and Type 2 Diabetes in PCOS: A Systematic Review and Meta‐Regression.” Human Reproduction Update 24, no. 4: 455–467. 10.1093/humupd/dmy007. 29590375

[fsn372007-bib-0027] Kałużna, M. , P. Kompf , K. Wachowiak‐Ochmańska , et al. 2022. “Are Patients With Polycystic Ovary Syndrome More Prone to Irritable Bowel Syndrome?” Endocrine Connections 11, no. 4: e210309. 10.1530/EC-21-0309. 35275093 PMC9066599

[fsn372007-bib-0028] Kogure, G. S. , V. B. Ribeiro , I. P. Lopes , et al. 2019. “Body Image and Its Relationships With Sexual Functioning, Anxiety, and Depression in Women With Polycystic Ovary Syndrome.” Journal of Affective Disorders 253: 385–393. 10.1016/j.jad.2019.05.006. 31082731

[fsn372007-bib-0029] Krug, I. , S. Giles , and C. Paganini . 2019. “Binge Eating in Patients With Polycystic Ovary Syndrome: Prevalence, Causes, and Management Strategies.” Neuropsychiatric Disease and Treatment 15: 1273–1285. 10.2147/NDT.S168944. 31190833 PMC6529622

[fsn372007-bib-0030] Labus, J. S. , R. Bolus , L. Chang , et al. 2004. “The Visceral Sensitivity Index: Development and Validation of a Gastrointestinal Symptom‐Specific Anxiety Scale.” Alimentary Pharmacology & Therapeutics 20, no. 1: 89–97. 10.1111/j.1365-2036.2004.02007.x. 15225175

[fsn372007-bib-0031] Lalonde‐Bester, S. , M. Malik , R. Masoumi , et al. 2024. “Prevalence and Etiology of Eating Disorders in Polycystic Ovary Syndrome: A Scoping Review.” Advances in Nutrition 15, no. 4: 100193. 10.1016/j.advnut.2024.100193. 38408541 PMC10973592

[fsn372007-bib-0032] Lane, M. M. , E. Gamage , N. Travica , et al. 2022. “Ultra‐Processed Food Consumption and Mental Health: A Systematic Review and Meta‐Analysis of Observational Studies.” Nutrients 14, no. 13: 2568. 10.3390/nu14132568. 35807749 PMC9268228

[fsn372007-bib-0033] Lee, I. , L. G. Cooney , S. Saini , et al. 2017. “Increased Risk of Disordered Eating in Polycystic Ovary Syndrome.” Fertility and Sterility 107, no. 3: 796–802. 10.1016/j.fertnstert.2016.12.014. 28104244

[fsn372007-bib-0034] Lim, S. S. , S. K. Hutchison , E. Van Ryswyk , R. J. Norman , H. J. Teede , and L. J. Moran . 2019. “Lifestyle Changes in Women With Polycystic Ovary Syndrome.” Cochrane Database of Systematic Reviews 2019, no. 3: CD007506. 10.1002/14651858.CD007506.pub4. PMC6438659 30921477

[fsn372007-bib-0035] Lupu, V. V. , C. M. Ghiciuc , G. Stefanescu , et al. 2023. “Emerging Role of the Gut Microbiome in Post‐Infectious Irritable Bowel Syndrome: A Literature Review.” World Journal of Gastroenterology 29, no. 21: 3241–3256. 10.3748/wjg.v29.i21.3241. 37377581 PMC10292139

[fsn372007-bib-0036] Martin, M. L. , K. Halling , D. Eek , M. Krohe , and J. Paty . 2017. “Understanding Polycystic Ovary Syndrome From the Patient Perspective: A Concept Elicitation Patient Interview Study.” Health and Quality of Life Outcomes 15, no. 1: 162. 10.1186/s12955-017-0736-3. 28821294 PMC5562990

[fsn372007-bib-0037] Martinez‐Perez, C. , L. Daimiel , C. Climent‐Mainar , et al. 2022. “Integrative Development of a Short Screening Questionnaire of Highly Processed Food Consumption (sQ‐HPF).” International Journal of Behavioral Nutrition and Physical Activity 19, no. 1: 6. 10.1186/s12966-021-01240-6. 35073909 PMC8785596

[fsn372007-bib-0038] Merhi, Z. 2019. “Crosstalk Between Advanced Glycation End Products and Vitamin D: A Compelling Paradigm for the Treatment of Ovarian Dysfunction in PCOS.” Molecular and Cellular Endocrinology 479: 20–26. 10.1016/j.mce.2018.08.010. 30170183

[fsn372007-bib-0039] Moghetti, P. , and F. Tosi . 2021. “Insulin Resistance and PCOS: Chicken or Egg?” Journal of Endocrinological Investigation 44, no. 2: 233–244. 10.1007/s40618-020-01351-0. 32648001

[fsn372007-bib-0040] Monteiro, C. A. , G. Cannon , J.‐C. Moubarac , R. B. Levy , M. L. C. Louzada , and P. C. Jaime . 2018. “The UN Decade of Nutrition, the NOVA Food Classification and the Trouble With Ultra‐Processing.” Public Health Nutrition 21, no. 1: 5–17. 10.1017/S1368980017000234. 28322183 PMC10261019

[fsn372007-bib-0041] Mulak, A. , Y. Taché , and M. Larauche . 2014. “Sex Hormones in the Modulation of Irritable Bowel Syndrome.” World Journal of Gastroenterology 20, no. 10: 2433–2448. 10.3748/wjg.v20.i10.2433. 24627581 PMC3949254

[fsn372007-bib-0042] Olszanecka‐Glinianowicz, M. , D. Dudek , K. J. Filipiak , et al. 2020. “Treatment of Overweight and Obesity During and After a Pandemic. Let's Not Wait for the Development of Complications—New Guidelines for Doctors.” Arterial Hypertension 24, no. 3: 93–105. 10.5603/AH.a2020.0019. 33740809

[fsn372007-bib-0043] Ozgen Saydam, B. , and B. O. Yildiz . 2021. “Polycystic Ovary Syndrome and Brain: An Update on Structural and Functional Studies.” Journal of Clinical Endocrinology and Metabolism 106, no. 2: e430–e441. 10.1210/clinem/dgaa843. 33205212

[fsn372007-bib-0044] Peters, M. , I. Mikeltadze , H. Karro , et al. 2022. “Endometriosis and Irritable Bowel Syndrome: Similarities and Differences in the Spectrum of Comorbidities.” Human Reproduction 37, no. 9: 2186–2196. 10.1093/humrep/deac140. 35713579

[fsn372007-bib-0045] Poursalehi, D. , S. A. Tirani , F. Shahdadian , Z. Hajhashemy , P. Rouhani , and P. Saneei . 2024. “Ultra‐Processed Foods Intake in Relation to Metabolic Health Status, Serum Brain‐Derived Neurotrophic Factor and Adropin Levels in Adults.” Nutrition Journal 23, no. 1: 121. 10.1186/s12937-024-01024-1. 39385201 PMC11462761

[fsn372007-bib-0046] Pujo, J. , G. De Palma , J. Lu , et al. 2023. “Gut Microbiota Modulates Visceral Sensitivity Through Calcitonin Gene‐Related Peptide (CGRP) Production.” Gut Microbes 15, no. 1: 2188874. 10.1080/19490976.2023.2188874. 36939195 PMC10038053

[fsn372007-bib-0047] Rodriguez Paris, V. , and M. J. Bertoldo . 2019. “The Mechanism of Androgen Actions in PCOS Etiology.” Medical Science 7, no. 9: 89. 10.3390/medsci7090089. PMC6780983 31466345

[fsn372007-bib-0048] Shishehgar, F. , F. Ramezani Tehrani , P. Mirmiran , S. Hajian , A. R. Baghestani , and N. Moslehi . 2016. “Comparison of Dietary Intake Between Polycystic Ovary Syndrome Women and Controls.” Global Journal of Health Science 8, no. 9: 302. 10.5539/gjhs.v8n9p302. PMC5064084 27157182

[fsn372007-bib-0049] Shoaibinobarian, N. , G. Eslamian , and M. Noormohammadi . 2022. “Association Between Ultra‐Processed Foods and Polycystic Ovary Syndrome: A Case‐Control Study.” 10.13140/RG.2.2.36013.97764. 33797335

[fsn372007-bib-0050] Shrivastava, R. , S. Patel , S. Mishra , R. Gupta , P. Shrivastava , and T. Pathak . 2024. “Effect of Yoga and Naturopathy on Sleep Quality and Pain in PCOS Patients With Primary Dysmenorrhea: A Case Series.” Integrative Medicine 23, no. 4: 29–32. 39355414 PMC11441583

[fsn372007-bib-0051] Song, Z. , R. Song , Y. Liu , Z. Wu , and X. Zhang . 2023. “Effects of Ultra‐Processed Foods on the Microbiota‐Gut‐Brain Axis: The Bread‐And‐Butter Issue.” Food Research International 167: 112730. 10.1016/j.foodres.2023.112730. 37087282

[fsn372007-bib-0052] Stefanaki, K. , D. S. Karagiannakis , M. Raftopoulou , T. Psaltopoulou , S. A. Paschou , and I. Ilias . 2023. “Obesity and Hyperandrogenism Are Implicated With Anxiety, Depression and Food Cravings in Women With Polycystic Ovary Syndrome.” Endocrine 82, no. 1: 201–208. 10.1007/s12020-023-03436-1. 37389719

[fsn372007-bib-0053] Sutcu, C. , G. Pamuk , and K. Ongel . 2021. “Evaluation of Night Eating Syndrome in Individuals With and Without Obesity.” Endokrynologia Polska 72, no. 5: 539–544. 10.5603/EP.a2021.0046. 34010444

[fsn372007-bib-0054] Szczuko, M. , M. Skowronek , M. Zapałowska‐Chwyć , and A. Starczewski . 2016. “Quantitative Assessment of Nutrition in Patients With Polycystic Ovary Syndrome (PCOS).” Roczniki Panstwowego Zakladu Higieny 67, no. 4: 419–426. 27925712

[fsn372007-bib-0055] Teede, H. J. , M. L. Misso , M. F. Costello , et al. 2018. “Recommendations From the International Evidence‐Based Guideline for the Assessment and Management of Polycystic Ovary Syndrome.” Fertility and Sterility 110, no. 3: 364–379. 10.1016/j.fertnstert.2018.05.004. 30033227 PMC6939856

[fsn372007-bib-0056] Teede, H. J. , C. T. Tay , J. Laven , et al. 2023. “Recommendations From the 2023 International Evidence‐Based Guideline for the Assessment and Management of Polycystic Ovary Syndrome.” Fertility and Sterility 120, no. 4: 767–793. 10.1016/j.fertnstert.2023.07.025. 37589624

[fsn372007-bib-0057] The Thessaloniki ESHRE/ASRM‐Sponsored PCOS Consensus Workshop Group . 2008. “Consensus on Infertility Treatment Related to Polycystic Ovary Syndrome.” Human Reproduction 23, no. 3: 462–477. 10.1093/humrep/dem426. 18308833

[fsn372007-bib-0058] Van Hooff, M. H. A. , F. J. Voorhorst , M. B. H. Kaptein , R. A. Hirasing , C. Koppenaal , and J. Schoemaker . 1999. “Endocrine Features of Polycystic Ovary Syndrome in a Random Population Sample of 14–16 Year Old Adolescents.” Human Reproduction 14, no. 9: 2223–2229. 10.1093/humrep/14.9.2223. 10469684

[fsn372007-bib-0059] Vincent, K. , C. Warnaby , C. J. Stagg , J. Moore , S. Kennedy , and I. Tracey . 2013. “Brain Imaging Reveals That Engagement of Descending Inhibitory Pain Pathways in Healthy Women in a Low Endogenous Estradiol State Varies With Testosterone.” Pain 154, no. 4: 515–524. 10.1016/j.pain.2012.11.016. 23318125

[fsn372007-bib-0060] WHO . 2025. “The Global Health Observatory Explore a World of Health Data.” https://www.who.int/data/gho/data/themes/topics/topic‐details/WHO/body‐mass‐index.

[fsn372007-bib-0061] Winters, S. J. , J. Gogineni , M. Karegar , et al. 2014. “Sex Hormone‐Binding Globulin Gene Expression and Insulin Resistance.” Journal of Clinical Endocrinology & Metabolism 99, no. 12: E2780–E2788. 10.1210/jc.2014-2640. 25226295

[fsn372007-bib-0062] Yang, Y. , H. Zhang , B.‐Y. Huang , et al. 2024. “Relationship Between Smoking, Excessive Androgen and Negative Emotions in Women With Polycystic Ovary Syndrome (PCOS).” Journal of Ovarian Research 17, no. 1: 211. 10.1186/s13048-024-01541-x. 39472904 PMC11520830

[fsn372007-bib-0063] Yüksel, S. , F. K. Gencer , F. B. Alptekin , and N. G. U. Saglam . 2024. “Disordered Eating in Young Women With Polycystic Ovary Syndrome.” Reproductive Sciences 31, no. 5: 1303–1310. 10.1007/s43032-023-01435-1. 38155280

[fsn372007-bib-0064] Zvolensky, M. , C. Jardin , S. G. Farris , et al. 2018. “Gut Interpretations: How Difficulties in Emotion Regulation May Help Explain the Relation of Visceral Sensitivity With Depression and Anxiety Among Young Adults With Gastrointestinal Symptoms.” Psychology, Health & Medicine 23, no. 7: 840–845. 10.1080/13548506.2018.1455984. PMC12826606 29580068

