# Supplementary material for: Associations of Ultra‐Processed Food Consumption and Night Eating With GI Symptom‐Specific Anxiety in Reproductive‐Age Women With Polycystic Ovary Syndrome
Source: Food Sci Nutr. 2026 Jun 10;14(6):e72007. doi: 10.1002/fsn3.72007 (PMC13253984; doi:10.1002/fsn3.72007)
Supplement: Supplementary file 1 — Table S1: Correlations among BMI, night eating, GI symptom‐specific anxiety, and ultra‐processed food consumption in women with PCOS. Table S2: Sensitivity analysis for GI symptom‐specific anxiety excluding BMI. Appendix S1: Participant informed consent form. [file FSN3-14-e72007-s001.docx]

**Supplementary Material**

**Supplementary Table 1.** Correlations Among BMI, Night Eating, GI Symptom-Specific Anxiety, and Ultra-Processed Food Consumption in Women with PCOS

| **Variable Pair** | **Whole Sample (n=205)** | **BMI<35 kg/m² Subgroup (n=182)** |
| --- | --- | --- |
| **BMI - NEQ** | r=0.282, p<0.001* | r=0.208, p=0.005* |
| **NEQ - VSI** | r=0.410, p<0.001* | r=0.406, p<0.001* |
| **VSI - UPF** | r=0.212, p=0.002* | r=0.218, p=0.003* |
| **BMI - VSI** | r=0.077, p=0.270 | r=0.113, p=0.128 |

Note: Data are presented as Pearson correlation coefficients (r) and p-values. BMI: Body Mass Index; NEQ: Night Eating Questionnaire total score; VSI: Visceral Sensitivity Index total score; UPF: Ultra-Processed Food consumption. **p < 0.05 indicates statistical significance. The BMI < 35 kg/m² subgroup represents a subset of the total sample (n = 182) to evaluate the consistency of associations.*

**Supplementary Table 2.** Sensitivity Analysis for GI Symptom-Specific Anxiety Excluding BMI

| **Independent Variables** | **Model 1: NEQ** | | | | |
| --- | --- | --- | --- | --- | --- |
|  | β | t | p | **95% CI** | **VIF** |
| **VSI total score** | 0.328 | 5.488 | <0.001* | 0.210, 0.446 | 1.08 |
| **sQ-HPF total score** | 0.118 | 1.937 | 0.054 | -0.002, 0.238 | 1.11 |
| **Smoking status** | 0.317 | 4.933 | <0.001* | 0.190, 0.443 | 1.24 |
| **Alcohol consumption** | 0.011 | 0.173 | 0.863 | -0.116, 0.138 | 1.25 |
| **Income status** | -0.170 | -2.861 | 0.005* | -0.287, -0.053 | 1.06 |
| **Marital status** | -0.092 | -1.550 | 0.123 | -0.210, 0.025 | 1.07 |
| **Model Statistics** | **R^2^=0.343, p<0.001** | | | | |
|  | **Model 2: VSI** | | | | |
|  | β | t | p | **95% CI** | **VIF** |
| **NEQ total score** | 0.402 | 5.488 | <0.001* | 0.258, 0.547 | 1.32 |
| **sQ-HPF total score** | 0.138 | 2.049 | 0.042* | 0.005, 0.270 | 1.11 |
| **Smoking status** | -0.108 | -1.444 | 0.150 | -0.256, 0.040 | 1.38 |
| **Alcohol consumption** | 0.009 | 0.119 | 0.905 | -0.132, 0.149 | 1.25 |
| **Income status** | -0.036 | -0.533 | 0.595 | -0.168, 0.096 | 1.10 |
| **Marital status** | -0.037 | -0.558 | 0.557 | -0.168, 0.094 | 1.08 |
| **Model Statistics** | **R^2^=0.195, p<0.001** | | | | |

Note: Data are presented as unstandardized coefficients (β), t-statistics, and p-values. 95% CI: 95% Confidence Interval; VIF: Variance Inflation Factor. Model 1 (Dependent variable: NEQ total score) and Model 2 (Dependent variable: VSI total score) were adjusted for age, ultra-processed food consumption, smoking status, alcohol consumption, income status, and marital status. *p < 0.05 indicates statistical significance. The sensitivity analysis was performed by excluding BMI (kg/m²) as a covariate to evaluate the stability of the associations.

**Supplementary Appendix A: Informed Consent Form**

You are being invited to participate in a research project. Before deciding whether to participate, it is essential for you to understand why and how the research will be conducted.

Participation in this study is strictly voluntary. Refusal to participate will not result in any penalty or loss of any benefits to which you are otherwise entitled. Similarly, even after agreeing to participate, you may withdraw from the research at any stage without providing any reason and without facing any harm or loss of expected benefits.

All financial expenses related to the procedures performed within the scope of the research will be covered by the researchers (Asst. Prof. Emine Merve EKİCİ) and will not impose any financial burden on you or your social security institution.

Please read the following information carefully and take some time to decide whether you wish to participate.

1. Study Title: Evaluation of the relationship between ultra-processed food consumption, visceral sensitivity symptoms, and night eating behavior in women with Polycystic Ovary Syndrome.

2. Principal Investigator: Asst. Prof. Emine Merve EKİCİ / University of Health Sciences, Gülhane Faculty of Health Sciences.

3. Purpose of the Study: This study aims to evaluate the association between ultra-processed food consumption, visceral sensitivity symptoms, and night eating behavior in women with Polycystic Ovary Syndrome.

4. Responsibilities of the Volunteer: You have no specific responsibilities other than completing the provided survey. You may leave the study at any time without providing any justification.

5. Risks and Discomforts: Pregnant women, postpartum women, and breastfeeding infants are excluded from the study.

6. Measures Against Risks: There will be no blood sampling or medical intervention. Participants will only complete a survey. Therefore, there are no physical risks involved.

7. Alternative Methods: Not applicable.

8. Compensation and Treatment for Research-Related Injury: Not applicable.

9. Payments for Transportation/Food: No payments will be made to volunteers for expenses such as transportation or meals.

10. Conditions for Termination of Participation: There is no specific condition requiring the termination of a volunteer's participation by the researchers; however, volunteers may withdraw at any time.

11. Information Post-Study: Volunteers will be informed about the results of the research upon request.

12. Contact Information: For further information about the research or in case of any unexpected events, you may contact the following investigator 24/7:

Asst. Prof. Emine Merve EKİCİ, Phone: +90 553 480 50 67

Statement of Consent

The purpose, investigators, and methodology of the study defined above have been explained to me in a language I can understand.

The potential benefits of this research to me and other people have been explained.

Possible risks and discomforts that may occur during the research have been described in an understandable language.

I have been provided with the name and phone number of an official whom I can contact 24 hours a day regarding potential side effects, risks, and my rights.

It has been explained that no fee will be requested from me or my social security institution for any examinations, tests, or medical care services within the scope of the research.

I am participating in this research voluntarily, without any pressure or coercion.

I have been informed that I have the right to refuse to participate.

I am aware that I can withdraw from this study at any time without providing any reason, provided that I inform the responsible researcher.

I know that my refusal to participate or subsequent withdrawal will not impose any responsibility on me and will not affect my current or future medical care.

I understand that the Gülhane Scientific Research Ethics Committee may, if deemed necessary, access my original medical records related to the research subject, in accordance with the principle of confidentiality.

It has been explained to me that my identity will be kept confidential in accordance with legal regulations and will not be disclosed to the public; research results may be presented at scientific meetings or published, but my identity will remain strictly confidential.

I have read this Informed Consent Form, which provides the necessary information before the study, in my native language. I was given the opportunity to ask all questions that came to my mind and received satisfactory answers. I agree to participate in this research under these conditions, voluntarily and without any pressure.

I have received a fully signed copy of the Informed Consent Form.

Volunteer’s (Handwritten):

Name-Surname: Signature:

Address (and Phone/Fax):

Date:

Researcher Conducting the Consent Process:

Name-Surname:

Signature:

Date:
